# MRI-Guided Motion-Corrected PET Image Reconstruction for Cardiac PET/MRI

**DOI:** 10.2967/jnumed.120.254235

**Published:** 2021-12

**Authors:** Camila Munoz, Sam Ellis, Stephan G. Nekolla, Karl P. Kunze, Teresa Vitadello, Radhouene Neji, Rene M. Botnar, Julia A. Schnabel, Andrew J. Reader, Claudia Prieto

**Affiliations:** 1 School of Biomedical Engineering and Imaging Sciences, King’s College London, London, United Kingdom;; 2 Nuklearmedizinische Klinik und Poliklinik, Technische Technical University of Munich, Munich, Germany;; 3 MR Research Collaborations, Siemens Healthcare, Frimley, United Kingdom;; 4 Department of Internal Medicine I, University Hospital Rechts der Isar, School of Medicine, Technical University of Munich, Munich, Germany; and; 5 Escuela de Ingeniería, Pontificia Universidad Católica de Chile, Santiago, Chile

**Keywords:** cardiac PET/MR, MR-guided PET reconstruction, MR-based motion correction

## Abstract

Simultaneous PET/MRI has shown potential for the comprehensive assessment of myocardial health from a single examination. Furthermore, MRI-derived respiratory motion information, when incorporated into the PET image reconstruction, has been shown to improve PET image quality. Separately, MRI-based anatomically guided PET image reconstruction has been shown to effectively denoise images, but this denoising has so far been demonstrated mainly in brain imaging. To date, the combined benefits of motion compensation and anatomic guidance have not been demonstrated for myocardial PET/MRI. This work addressed this lack by proposing a single cardiac PET/MR image reconstruction framework that fully utilizes MRI-derived information to allow both motion compensation and anatomic guidance within the reconstruction. **Methods:** Fifteen patients underwent an ^18^F-FDG cardiac PET/MRI scan with a previously introduced acquisition framework. The MRI data processing and image reconstruction pipeline produces respiratory motion fields and a high-resolution respiratory motion–corrected MR image with good tissue contrast. This MRI-derived information was then included in a respiratory motion–corrected, cardiac-gated, anatomically guided image reconstruction of the simultaneously acquired PET data. Reconstructions were evaluated by measuring myocardial contrast and noise and were compared with images from several comparative intermediate methods using the components of the proposed framework separately. **Results:** Including respiratory motion correction, cardiac gating, and anatomic guidance significantly increased contrast. In particular, myocardium–to–blood pool contrast increased by 143% on average (*P* < 0.0001), compared with conventional uncorrected, nonguided PET images. Furthermore, anatomic guidance significantly reduced image noise, by 16.1%, compared with nonguided image reconstruction (*P* < 0.0001). **Conclusion:** The proposed framework for MRI-derived motion compensation and anatomic guidance of cardiac PET data significantly improved image quality compared with alternative reconstruction methods. Each component of the reconstruction pipeline had a positive impact on the final image quality. These improvements have the potential to improve clinical interpretability and diagnosis based on cardiac PET/MR images.

## Visual Abstract

**Figure:**
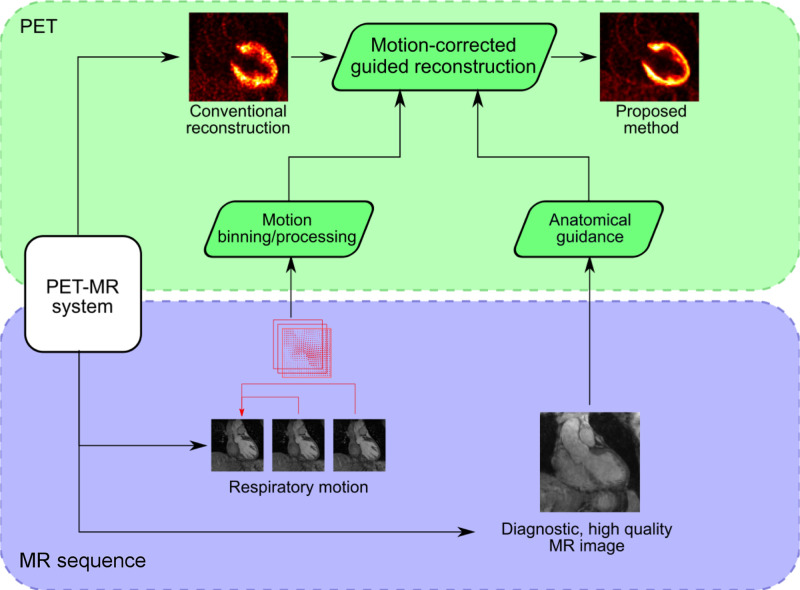

Simultaneous PET/MRI allows the comprehensive assessment of cardiovascular disease. Nearly a decade after the introduction of hybrid PET/MRI systems, several studies focusing on the assessment of myocardial viability and inflammatory and infiltrative diseases have shown the benefit of the complementary functional and anatomic information provided by both imaging modalities (*1–3*).

PET/MRI has also opened new possibilities for addressing several of the technical challenges that may affect PET image quality. Accurate attenuation correction is fundamental to clinical interpretability and quantification of cardiac PET data; however, attenuation maps (μ-maps) are typically acquired during a breath-hold before the actual PET acquisition, resulting in a potential misalignment between the μ-map and the PET image position. This misalignment may lead to artifacts that appear as reduced myocardial uptake and could be mistaken for myocardial defects (*4*). To improve the correspondence between attenuation and emission data, specialized MRI acquisition schemes have been proposed to enable free-breathing (*5*) or respiratory-resolved μ-maps (*6*).

Another source of image degradation is physiologic (i.e., respiratory and cardiac) motion throughout a PET data acquisition; this motion may induce blurring in the final images if not accounted for. Several proposed approaches toward MRI-based PET motion compensation can estimate and correct for organ displacement due to physiologic motion by simultaneously acquiring dynamic MR images with sufficient tissue contrast (*7*–*9*). These techniques have shown promising results for improving PET image quality, but most are limited in that the MR images simultaneously acquired with PET data are designed to be used for motion estimation only. The insufficient spatial resolution or appropriate tissue contrast of these MR images limits their use for diagnostic purposes and hinders the full realization of the potential of truly simultaneous cardiac PET/MRI.

Finally, the count-limited nature of PET image acquisition causes noise in the reconstructed images. In conventional PET image reconstruction, such as maximum-likelihood expectation maximization (MLEM) (*10*), this noise increases with the number of iterations, and reconstructions are therefore usually terminated early. However, early termination results in bias due to underconvergence, reducing the quantitative value of the reconstructed PET images. In the context of PET/MRI, anatomic information provided by the simultaneously acquired MR images can be used to guide the PET image reconstruction, enabling noise reduction and partial-volume correction (*11*,*12*). Although these approaches have shown significant improvements in brain PET/MRI, their use in cardiac PET/MRI applications has not been explored so far, because enabling accurate anatomic guidance from cardiac MR images requires 3-dimensional images that have sufficient volumetric coverage and high tissue contrast and that also provide information about physiologic motion (so they can be motion-aligned to the PET image position). The acquisition of such images is challenging, as most clinically available cardiac MRI protocols are based on acquiring stacks of 2-dimensional slices under repeated breath-holds.

Here, we introduce a single framework that exploits the advantages of cardiac PET/MRI to improve myocardial PET imaging by integrating several elements of state-of-the-art PET image reconstruction. We used a recently introduced cardiac PET/MRI protocol designed for simultaneous diagnostic PET and coronary MR angiography (CMRA) (*13*), which provides both respiratory motion information and a whole-heart high-resolution CMRA image that allows for myocardial PET image reconstruction to be improved as follows: first, μ-maps are aligned to the end-expiration respiratory position using the CMRA images as a reference to reduce attenuation-induced artifacts; second, MRI-derived motion information is incorporated into a motion-corrected image reconstruction of the PET data; and third, high-contrast motion-corrected 3-dimensional CMRA images are used for anatomically guided PET image reconstruction, suppressing noise while preserving quantification performance. We tested the proposed framework in a small cohort of patients without cardiac disease to quantify the effect of each of these improvements on final image quality, including myocardium-to-blood contrast and noise levels. Furthermore, we applied the framework to a cohort of 10 patients with cardiac disease, showing that the proposed method achieves visually superior images compared with conventional PET image reconstruction.

## MATERIALS AND METHODS

**FIGURE 1.**
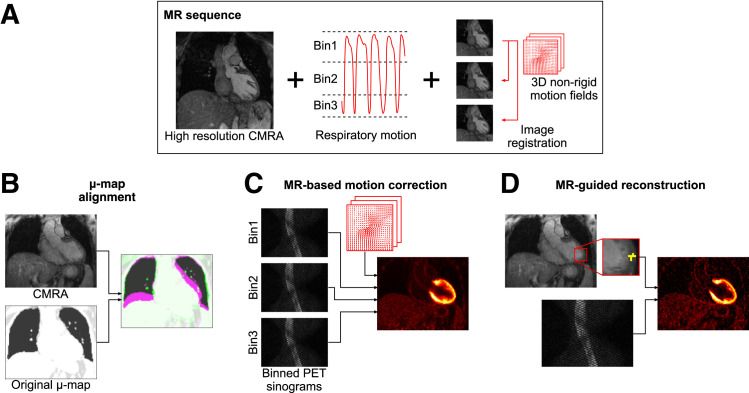
Overview of MRI-based improvements for proposed PET image reconstruction framework. MRI sequence provides high-quality end-expiration CMRA image and respiratory motion information (A), which can be used to improve PET image reconstruction by aligning PET μ-maps to CMRA position (B), performing MRI-based motion-correction (C), and performing MRI-guided PET image reconstruction (D).

**FIGURE 2.**
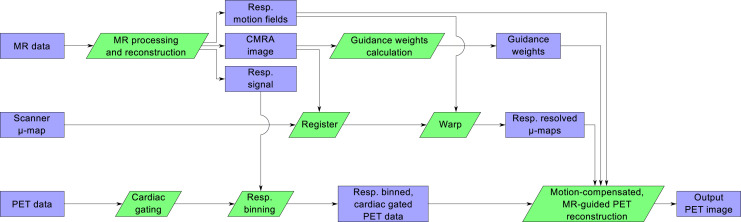
Flowchart of proposed PET/MR image reconstruction pipeline.

The proposed framework uses a previously introduced cardiac PET/MRI sequence (*13*) to produce high-quality whole-heart MRI information that allows for accurate alignment of PET μ-maps, estimation of respiratory motion fields for motion-compensated PET image reconstruction, and anatomically guided PET image reconstruction (Fig. 1). Each component of the proposed framework is described below, and a flowchart of the entire pipeline is shown in Figure 2.

### MRI-Based Respiratory Motion Correction and μ-Map Alignment

The cardiac MR image reconstruction process produces 3-dimensional MR images in various respiratory states as an intermediate output, which is used to estimate respiration-induced motion throughout the breathing cycle. This respiratory motion information can be used to correct the concurrent PET data by grouping them into corresponding respiratory bins and performing a motion-compensated PET image reconstruction (*14*) using the same motion fields as for the MR image reconstruction. Similar to the cardiac MR image, the output of this PET image reconstruction is an end-expiration cardiac PET image. To minimize the effect of cardiac motion (i.e., motion due to the heart beating), about 30% of the PET data corresponding to systole are discarded using the electrocardiogram signal as a reference.

To improve the correspondence between the μ-map and the PET image position, the conventional breath-held μ-map is registered to the end-expiration CMRA image before PET image reconstruction. Nonrigid registration is performed using a free-form deformation algorithm with a normalized mutual-information objective function (*15*).

### MRI-Guided PET Image Reconstruction

The high-resolution CMRA images are then used to perform patient-specific anatomically guided PET image reconstructions. Anatomic guidance groups voxels that are expected to have a similar PET intensity (e.g., due to being in close proximity to each other and composed of the same tissue type) and applies anisotropic smoothing between these voxels.

The proposed framework uses a weighted quadratic penalty (*16*), wherein the a priori similarity between a PET voxel *j* and each of its neighboring voxels *k* is calculated from the MR image as the patch-based Euclidean distance modulated by a gaussian kernel:

$$w_{jk}=\exp\;\left(\frac{-f_{j}-f_{k}{}^{2}}{2{\text{σ}}^{2}}\right)\;\forall k\in N_{j},$$

where $f_{j}$ is the MRI patch centered at voxel *j*, $N_{j}$ is the neighborhood around voxel *j*, and σ controls the width of the gaussian similarity kernel. In this work, $f_{j}$ is a 3 × 3 × 3 voxel patch, with $N_{j}$ also set to 3 × 3 × 3, and σ equals 0.1 (with MR images normalized between 0 and 1). Furthermore, as previously suggested (*16*), only weights corresponding to the 7 most similar neighbors for each voxel are kept, with all other weights set to 0.

These similarity weights are calculated for each patient and incorporated into the PET image reconstructions using a modified maximum a posteriori expectation maximization (MAPEM) algorithm (*17*). To avoid relying on user-specified regularization strengths, a recently proposed method for automatic setting of this value (*18*) was used. The regularized reconstructions were run for 200 iterations using the patient-specific automatically selected regularization strength.

### Experiments

#### Data Acquisition

PET/MRI data were acquired for 15 patients (mean age ± SD, 62.8 ± 12.5 y; 11 male, 4 female) with a simultaneous PET-CMRA sequence (13) after injection of 331.3 ± 27.9 MBq of ^18^F-FDG. Full details of the CMRA acquisition were previously published (*13*).

Two patient cohorts were included in this study. The first cohort included 5 oncology patients without known or suspected cardiovascular disease, who exhibited physiologic myocardial uptake of ^18^F-FDG. In the absence of cardiac conditions, ^18^F-FDG uptake is expected to be uniform throughout the myocardium. These patients underwent a clinical PET/CT examination and were subsequently scanned in a PET/MRI scanner without additional radiotracer administration. These data have previously been used to demonstrate a respiratory motion compensation scheme for cardiac PET (*13*).

The second cohort comprised 10 patients with symptomatic coronary artery disease, chronic total occlusion of a relevant coronary artery, and evidence of wall motion abnormalities. These patients underwent a clinical PET/MRI examination protocol for the assessment of myocardial viability, which included a 40- to 50-min list-mode PET acquisition using ^18^F-FDG under insulin-clamped conditions and conventional 2-dimensional late gadolinium-enhanced MRI. The data have previously been published in a clinical validation of respiratory motion compensation (19). Compared with previous work, the current study introduced a new PET image reconstruction framework that integrates μ-map alignment, respiratory motion correction, cardiac gating, and MRI guidance to further improve image quality.

All acquisitions were performed on a Biograph mMR scanner (Siemens Healthcare). All subjects gave written informed consent, and the study was performed in concordance with the Declaration of Helsinki and approved by the corresponding Institutional Ethics Committee.

#### Comparative Methods

To assess the effect of each component of the proposed cardiac PET image reconstruction method, various comparative reconstruction methods were performed:

Clinically representative reconstructions of the PET datasets were performed using MLEM (*10*). These reconstructions do not include respiratory motion correction (no motion-corrected, NMC) and were run for a clinically representative 63 iterations (NMC-MLEM-63). To assess the effect of μ-map alignment, the same reconstructions were run using μ-maps registered to the end-expiration CMRA images (NMC-MLEM-63-μ-reg). The NMC-MLEM reconstruction with a registered μ-map was also run for 200 iterations to investigate the effect of convergence (NMC-MLEM-200-μ-reg).

Respiratory motion–corrected (MC) MLEM reconstructions were performed, using the registered μ-map, without anatomic guidance (MC-MLEM-200-μ-reg). These reconstructions were also run to convergence, that is, 200 iterations. To investigate the effect of cardiac motion, MC (respiratory motion-corrected) image reconstructions were performed using cardiac-gated data (MC-MLEM-200-μ-reg-gated), with data acquired during systole being rejected as described above.

Finally, a reconstruction with the complete proposed method was performed, incorporating aligned μ-maps, respiratory motion correction, cardiac gating, and anatomic guidance with automatic regularization strength selection (MC-guided-MAPEM-μ-reg-gated).

All PET image reconstructions were performed in MATLAB (MathWorks) with custom-developed software (*20*). Relevant PET reconstruction parameters include resolution modeling (4.3 mm in full width at half maximum [FWHM]), a voxel size of 2.03 × 2.08 × 2.08 mm, and a matrix size of 127 × 344 × 344. All list-mode PET data were truncated to the scan duration of the MRI sequence to allow use of the MRI respiratory trace for PET data binning.

### Image Analysis

PET image quality was analyzed in terms of noise and contrast. Contrast was measured as the contrast recovery coefficient (CRC) between the left-ventricular myocardium and the blood pool, and noise was calculated as the SD of the voxels within the myocardium. To obtain the myocardial and blood pool regions, the CMRA images were semiautomatically segmented using 3DSlicer (*21*). To assess the local effect of motion compensation, gaussian curves were fitted to 3 profiles through the left ventricular myocardium for each oncology patient, with the estimated FWHM serving as a surrogate for myocardial sharpness. All metrics were compared between reconstruction methods using a paired 2-tailed Student *t* test, with a *P* value of less than 0.05 considered to indicate a statistically significant difference.

Additionally, 17-segment analysis (*22*) was performed to assess the impact of the proposed quantification method at a segment level, and contrast between healthy myocardium and myocardial viability defects was computed from manually defined regions of interest in patients with transmural defects.

## RESULTS

**FIGURE 3.**
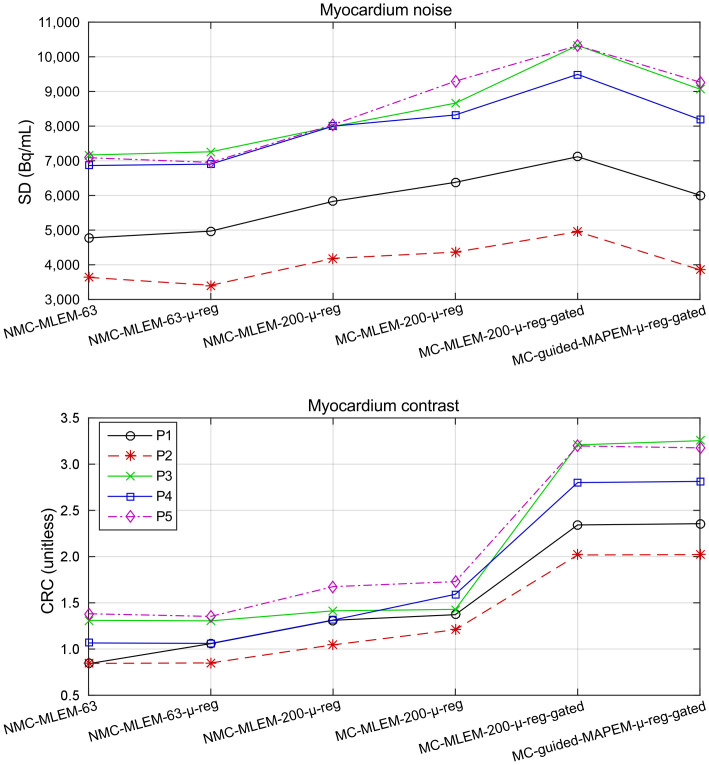
Image noise and contrast in 5 oncology patients for each PET image reconstruction method. Proposed method provides highest myocardium-to-blood pool contrast levels (CRC) in all cases while avoiding high noise levels (SD) of unregularized PET image reconstruction.

**FIGURE 4.**
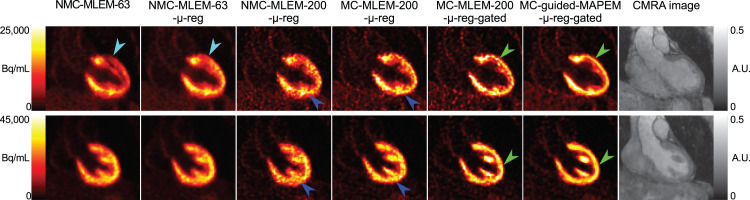
Reconstructed images for each comparative method for 2 representative oncology patients (1 per row). Cyan arrows indicate myocardial defect-mimicking attenuation artifact, which is removed by aligning μ-map using CMRA image. Blue arrows highlight improved local contrast when using motion-corrected PET image reconstruction. Green arrows show reduced noise and improved sharpness when combining motion compensation with MRI-guided PET image reconstruction.

Figure 3 shows the effect of each element of the proposed method in terms of CRC and myocardium SD for the first patient cohort, and Figure 4 demonstrates these differences in the reconstructed images for 2 representative patients.

Correction of PET μ-maps was seen to have no strong impact on myocardium SD in any of the patients. In terms of CRC, 4 of the 5 patients showed no change; however, in patient 1, an increase in CRC was apparent. This increase was due to a particularly poor alignment between the average free-breathing end-expiration position and the position of the breath-hold μ-map, leading to a defect-mimicking artifact that was alleviated by aligning the μ-map (Fig. 4).

With a correctly aligned μ-map, increasing the number of MLEM iterations from 63 to 200 increased the myocardium SD and CRC in all cases. These effects are due to the convergence of the MLEM algorithm; improved contrast represents convergence toward true regional means, whereas increased noise at convergence is a well-known characteristic of MLEM reconstructions.

When motion correction is included in the form of MC-MLEM-200-μ-reg, contrast and noise both increase further because of the deblurring effect of motion correction (Fig. 4) and the increased noise arising from the MC-MLEM algorithm, as has previously been shown in the literature (*7*,*23*,*24*). Performing cardiac gating of PET images using MC-MLEM-200-μ-reg-gated greatly increases myocardial CRC, because of the removal of blurring artifacts from cardiac motion. However, noise also increases since reducing counts from the PET data (by rejecting systolic data) results in a lower signal-to-noise ratio. Finally, by including MRI guidance in the motion-corrected, cardiac-gated PET reconstruction, CRC generally remains at similar or slightly higher levels while noise reduces.

**FIGURE 5.**
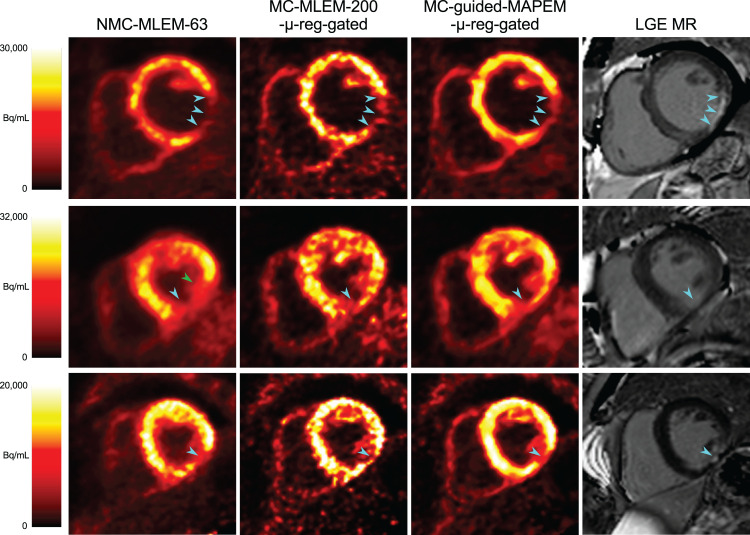
Example short-axis view of reconstructed ^18^F-FDG PET images for 3 selected CTO patients, and corresponding late-gadolinium enhancement (LGE) MR images, showing extent of myocardial scarring. Proposed method improves image quality while maintaining appearance of ^18^F-FDG hypointense defects (cyan arrowheads). MC-MLEM-200-μ-reg-gated images are shown to distinguish effects of motion compensation and guidance. In some cases, uncorrected PET images falsely depict defect as more extensive than it actually is (green arrowhead). LGE images are shown only for comparison and did not provide any information for guided PET reconstructions, which instead used high-resolution CMRA images.

Similar trends were observed for the patients with chronic total occlusion. Figure 5 shows example PET images reconstructed with the conventional NMC-MLEM-63 and MC-MLEM-200-μ-reg-gated methods, and the proposed MC-guided-MAPEM-μ-reg-gated method, alongside reference late gadolinium-enhanced MR images highlighting regions of myocardial scarring. The previously described improvements in noise and contrast are visible, and the depiction of myocardial defects (hypointense regions) is preserved, coinciding with the hyperintense regions in the late gadolinium-enhanced MR images.

The proposed MC-guided-MAPEM-μ-reg-gated method increases the CRC of transmural defects by 18.6% and their contrast-to-noise ratio by 47.7% (Supplemental Fig. 1; supplemental materials are available at http://jnm.snmjournals.org), indicating that the MRI guidance information is sufficiently localized to maintain relevant patterns of uptake while successfully reducing noise.

Summary statistics for both CRC and SD for all patients are shown in Table 1. Each incremental improvement in the PET image reconstruction method increased CRC significantly, except when introducing motion correction (*P* = 0.078). For myocardial SD, all differences were significant, except for alignment of the μ-map (*P* = 0.20). The only comparison in which SD decreased significantly was when introducing MRI guidance to the MC-MLEM reconstruction. Furthermore, 17-segment analysis indicated that all segments showed an increased uptake with the proposed method, which ranged from about a 15% increase at the apical segments to more than a 60% increase toward the basal anterior segments (Supplemental Fig. 2).

**TABLE 1:** Statistical Analysis of Effect of Comparative Methods in Terms of CRC and SD for Combined Patient Cohort (Chronic Total Occlusion Plus Oncology)

	$\bar{\text{CRC}}$ (unitless)		$\bar{\text{SD}}$ (Bq/mL)	
Combined cohort (*n* = 15)	Data	*P*	Data	*P*
NMC-MLEM-63	0.92 ± 0.30		5,443 ± 1,636	
NMC-MLEM-63-μ-reg	0.96 ± 0.30	0.033 (↑)	5,512 ± 1,660	0.20 (↑)
NMC-MLEM-200-μ-reg	1.23 ± 0.39	8.2 × 10^−7^ (↑)	6,565 ± 1,805	1.2 × 10^−8^ (↑)
MC-MLEM-200-μ-reg	1.34 ± 0.44	0.078 (↑)	7,033 ± 1,974	5.0 × 10^−5^ (↑)
MC-MLEM-200-μ-reg-gated	2.22 ± 0.72	1.1 × 10^−5^ (↑)	7,752 ± 2,302	3.7 × 10^−5^ (↑)
MC-guided-MAPEM-μ-reg-gated	2.24 ± 0.73	0.0026 (↑)	6,504 ± 2,237	1.7 × 10^−8^ (↓)
Comparison*		1.2 × 10^−7^ (↑)		2.8 × 10^−5^ (↑)

* *P* values comparing proposed method with clinical standard NMC-MLEM-63.

*P* values from 2-tailed paired *t* tests are shown, along with sign of change (↑ for positive, ↓ for negative), comparing method in each row with previous row.

In terms of myocardial sharpness, a similar trend could be observed (Supplemental Table 1), with the proposed MC-guided-MAPEM-μ-reg-gated method reducing FWHM by 24.6% ± 13.9% compared with the conventional NMC-MLEM-63, and MC-MLEM-200-μ-reg-gated images reducing FWHM by 26.6% ± 16.9%, with no statistically significant difference between the 2 methods (*P* = 0.27).

## DISCUSSION

The aim of this study was to introduce a framework for motion-corrected, MRI-guided PET image reconstruction for myocardial PET/MRI and demonstrate the capabilities of the proposed method to improve PET image quality by increasing contrast without introducing excessive noise into the output images.

A series of incrementally improved PET image reconstruction approaches was compared for 2 cohorts of patients, with and without known cardiac disease. Quantitatively, each of the incremental improvements showed increased contrast (Fig. 3); however, this was generally at the cost of additional noise. Compared with the MC-MLEM-200-μ-reg-gated reconstruction, the proposed method incorporating MRI guidance further improved contrast while reducing noise. Statistical analysis showed that these improvements were generally statistically significant, with the notable exception of the MC reconstruction.

This lack of significance when including motion correction may be due to both the nonrigid nature of the respiration-induced motion of the heart and the variability in respiratory patterns across different patients. Although motion correction can demonstrate strong improvement in areas of high motion (e.g., Fig. 4), areas of reduced motion in patients with shallower breathing patterns will result in less difference between corrected and uncorrected images.

The proposed method did not reduce noise down to clinical standard levels (Table 1). However, noise levels in clinical PET images (i.e., at 63 iterations) are arbitrary since sufficiently early termination can provide almost arbitrarily low noise. Similarly, the proposed method could achieve additional noise reduction by manually varying the regularization strength. This manually selected regularization would still lead to increased CRC at matched noise levels, although a comprehensive evaluation of this approach remains for future work. Furthermore, the automatic regularization selection method is designed to be error-optimal, rather than noise-optimal (*18*). When the guidance information is imperfect, regularization strength will be reduced in order to produce a faithful, if more noisy, representation of the PET data. For this reason, the noise levels from the proposed reconstruction method are a function of both raw PET data noise level and accuracy of correspondence between the MR and PET images. By producing better MRI-based guidance information, or modeling uncertainties of guidance information, regularization strength could be increased, further reducing noise.

Despite the promising results demonstrated in this study, there are several areas for improvement that could be addressed in future work.

The proposed reconstruction method requires the selection of several tunable hyperparameters, including motion estimation (number of respiratory bins, image registration parameters) and anatomic guidance parameters ($N_{j}$, σ). In this work, such parameters were selected as in previous studies, and their impact on the final image quality was not studied. Future work includes a comprehensive investigation of these hyperparameters, which would be required before using the proposed method in a clinical setting. Furthermore, the proposed reconstruction is slow, requiring about 10 h of computing time per patient, using a single Intel Xeon 2.6-GHz central processing unit with an NVIDIA Tesla K40 M graphics processing unit for the PET projection operators—resulting in a reconstruction time that is impractical for clinical adoption. Options for acceleration include pursuing a subset-based implementation (*25*) or applying the MRI guidance after reconstruction with a deep-learning approach (*26*).

Although this study demonstrated improvements in contrast and noise (considering the whole myocardium) and highlighted correspondence between PET and late gadolinium-enhanced images, and although preliminary assessment showed that the proposed method increased the contrast-to-noise ratio for transmural defects, the clinical utility of the images produced by the proposed method in terms of detection and assessment of myocardial defects was not thoroughly evaluated. Further studies in which the detectability and delineation of myocardial viability defects are assessed by expert observers in a larger cohort of patients are required to fully evaluate the diagnostic value of this technique.

In clinical practice, cardiac PET/MRI protocols can be up to 40 min long, but the proposed framework uses approximately only 10 min of simultaneous PET-CMRA data. The method could be extended to include longer PET acquisitions by using surrogate respiratory motion signals (*8*).

Although the PET and MRI data in this study were simultaneously acquired, there remains no guarantee that the positions of the 2 images correspond perfectly. System imperfections could lead to misalignment between imaging modalities. Additionally, the diastolic PET acquisition window in each heartbeat is much longer than the MRI acquisition window, potentially leading to residual cardiac motion in the PET data that could cause misalignment between the PET and MR images. This motion could be alleviated by gating the PET data even more restrictively. However, more restrictive gating would reduce the counts in the PET data further, potentially impacting image quality. Alternatively, this cardiac motion–induced misalignment could be estimated during the reconstruction (*27*), although this approach would add considerable computational cost.

## CONCLUSION

Simultaneous PET/MRI has shown potential for improving PET image quality by using MRI information to address various degrading factors. In separate research works, MRI-based motion compensation and MRI-guided reconstruction have been demonstrated to improve PET image quality. In this work, these developments were integrated into a single PET/MRI framework that produces high-quality, diagnostic CMRA images alongside improved ^18^F-FDG cardiac PET images. The proposed integrated PET image reconstruction framework improves image quality by including MRI-based μ-map alignment, respiratory motion correction and cardiac gating, and MRI-based guidance of PET data.

The proposed framework was evaluated in terms of contrast and noise and was compared with several alternative reconstruction methods for each component of the PET reconstruction approach. The proposed method produced the highest contrast of all the methods and significantly reduced image noise compared with a reference reconstruction that incorporates the motion compensation components of the framework without MRI guidance. In addition, applying the proposed framework to ^18^F-FDG PET data from cardiac patients demonstrated that the visual appearance of clinically relevant features, such as hypointense defects, are preserved.

## DISCLOSURE

This work was supported by the following grants: EPSRC EP/P032311/1, EP/P007619/1, EP/P001009/1, BHF program grant RG/20/1/34802, and WT 203148/Z/16/Z from the Wellcome/EPSRC Centre for Medical Engineering. This research was also supported by the National Institute for Health Research (NIHR) Cardiovascular Health Technology Cooperative (HTC) and the Biomedical Research Centre based at Guy’s and St. Thomas’ NHS Foundation Trust and King’s College London. No other potential conflict of interest relevant to this article was reported.

## Notes

KEY POINTS **QUESTION:** What is the impact on image quality of incorporating MRI-based motion compensation and anatomic guidance in cardiac PET image reconstruction? **PERTINENT FINDINGS:** The proposed method was tested in 2 cohorts of patients with myocardial uptake who underwent PET/MRI examinations. The proposed PET reconstruction approach showed a significant increase in myocardium–to–blood pool contrast compared with standard images, indicative of improved PET quantification. Compared with a converged PET image reconstruction method, inclusion of MRI guidance significantly reduced noise while preserving edges. **IMPLICATIONS FOR PATIENT CARE:** The improved myocardial PET image quality obtained by the proposed method has the potential to enhance diagnostic imaging by providing more accurate cardiac PET images.
